# Factors Associated with Non-Severe Adverse Reactions after Vaccination against SARS-CoV-2: A Cohort Study of 908,869 Outpatient Vaccinations in Germany

**DOI:** 10.3390/vaccines10040566

**Published:** 2022-04-06

**Authors:** Sven H. Loosen, Jens Bohlken, Kerstin Weber, Marcel Konrad, Tom Luedde, Christoph Roderburg, Karel Kostev

**Affiliations:** 1 Clinic for Gastroenterology, Hepatology and Infectious Diseases, University Hospital Düsseldorf, Medical Faculty of Heinrich Heine University Düsseldorf, 40225 Düsseldorf, Germany; luedde@hhu.de; 2 Institut für Sozialmedizin, Arbeitsmedizin und Public Health (ISAP), Medical Faculty, University of Leipzig, 04103 Leipzig, Germany; dr.j.bohlken@gmx.net; 3 Impfzentrum Messe 21 Berlin, 14055 Berlin, Germany; kerstinweber.berlin@t-online.de; 4 Health & Social, FOM University of Applied Sciences for Economics and Management, 60549 Frankfurt, Germany; konrad.marcel@web.de; 5 Epidemiology, IQVIA, 60549 Frankfurt, Germany; karel.kostev@iqvia.com

**Keywords:** COVID-19, SARS-CoV-2, Comirnaty, BioNTech, Moderna, side effect, mRNA, vector

## Abstract

Background: Vaccination against SARS-CoV-2 significantly reduces the transmissibility of the virus and the likelihood of a severe course of COVID-19, and is thus a critical component in overcoming the current pandemic. The factors associated with adverse reactions after vaccination against SARS-CoV-2 have not yet been sufficiently evaluated. Methods: We used the Disease Analyzer database (IQVIA) to identify 531,468 individuals who received a total of 908,869 SARS-CoV-2 vaccinations in 827 general practices in Germany between April and September 2021. Cox regression models were used to analyze the frequency of vaccination-related side effects reported within 14 days after SARS-CoV-2 vaccination, as well as subjects’ demographic characteristics and comorbidities. Results: The total number of side effects documented was 28,287 (3.1% of all vaccinations). Pain in the limb (24.3%), fatigue (21.0%), dizziness (17.9%), joint pain (15.7%), fever (9.5%), nausea (7.5%), and myalgia (6.4%) were the most common side effects documented among the 12,575 vaccinations with definite side effects. In the multivariate regression analysis, young age was associated with much higher odds of reported side effects (OR_18–30 years_: 4.45, OR_31–40 years_: 3.50, OR_41–50 years_: 2.89). In addition, pre-existing comorbidities such as dementia (OR: 1.54), somatoform disorder (OR: 1.53), anxiety disorder (OR: 1.43), depression (OR: 1.37), chronic respiratory tract disease (OR: 1.27), hypertension (OR: 1.20), and obesity (1.14) significantly increased the odds of side effects. Finally, the male sex was associated with increased odds of reported side effects (OR: 1.17). Conclusion: Our study, based on a large outpatient database from Germany, identified young age, male sex, and pre-existing comorbidities such as dementia, somatoform disorders, anxiety disorders, and depression as factors associated with vaccine-related adverse events diagnosed in GP practices. These data could help to identify subgroups needing particular advice and care in the context of SARS-CoV-2 vaccinations.

## 1. Introduction

The current SARS-CoV-2 pandemic is a major global challenge that is critically impacting multiple sectors of daily life and is still associated with high levels of morbidity and mortality [1]. The extremely rapid development of multiple vaccines against SARS-CoV-2 within just one year made it possible to vaccinate a large part of the world’s population in national vaccination campaigns, decisively changing the course of the pandemic in a positive way and preventing many SARS-CoV-2 deaths [2,3,4]. While the vaccines available are generally well tolerated and have a low number of side effects [2,3,4,5], there is still a fear of possible side effects among some of the population worldwide, which means that the target vaccination rates of, e.g., more than 90%, often cannot be achieved.

For the vaccines mainly administered in Germany, i.e., the two mRNA vaccines “Comirnaty” (BioNTech, Pfizer) and “Spikevax” (Moderna), as well as the vector vaccine “Vaxzevria” (AstraZeneca), the registration trials describe pain at the injection site, fatigue, and headache as the most common side effects [2,3,4,5]. Vaccinators discuss these common side effects with patients with the help of a standardized information sheet before administering the vaccine. The doctor and patient must confirm in writing on the information sheet that this information has been provided. For example, the Comirnaty information sheet states that adverse reactions are less common in older people than in younger people. Comirnaty registration studies report nausea, redness at the site of injection in fewer than 10% of subjects, and lymph node swelling, insomnia, malaise, itching, or hypersensitivity reactions in the vaccination arm in fewer than 1%. However, there are only limited population-based data that identifies possible clinical risk factors for the reporting of side effects after vaccination against SARS-CoV-2. Given the large number of patients that have already received such vaccinations and the even higher number of patients that will receive a SARS-CoV-2 vaccination in the near future, such data are of tremendous relevance. Therefore, in the present study, we analyzed the epidemiology of non-severe adverse reactions following outpatient SARS-CoV-2 vaccination in the real-life setting.

## 2. Materials and Methods

### 2.1. Database

This study was based on data from the Disease Analyzer database (IQVIA), which contains drug prescriptions, diagnoses, and basic medical and demographic data obtained directly and in anonymous format from computer systems used in the practices of general practitioners and specialists [6]. The database covers approximately 3% of all outpatient practices in Germany. Diagnoses (according to the International Classification of Diseases, 10th revision [ICD-10]), prescriptions (according to the Anatomical Therapeutic Chemical [ATC] classification system), and the quality of reported data are monitored continuously by IQVIA. In Germany, the sampling methods used to select physicians’ practices are appropriate for obtaining a representative database of general and specialized practices. It has previously been shown that the panel of practices included in the Disease Analyzer database is representative of general and specialized practices in Germany [6]. Finally, this database has already been used in previous studies focusing on SARS-CoV-2 [7,8,9], specifically the psychological consequences of SARS-CoV-2 [10,11]. The “Disease Analyzer” database used for analysis contains anonymized electronic patient records. Patient data were analyzed in aggregated form and no individual data were available. Therefore, in line with national and European legislation, no individual consent forms were required or obtained.

### 2.2. Study Population and Study Outcome

This retrospective cohort study included individuals aged ≥18 with an initial vaccination for COVID-19 given in 827 general practices in Germany between April 2021 and September 2021. The outcome of the study was the frequency of documented vaccination side effects within 14 days after SARS-CoV-2 vaccination (ICD-10: U12.9) depending on age, sex, and defined comorbidities (see Table 1 for details). For patients who received two or three doses each, the number of vaccinations was included separately in the analyses.

### 2.3. Statistical Analyses

A multivariate logistic regression model was applied to evaluate the association between predefined variables and the odds of reported side effects. The dependent variable was a documentation of side effect (yes/no); independent variables included age group (18–30, 31–40, 41–50, 51–60, 61–70, and >70 years), sex, and comorbidities documented within 12 months prior to SARS-CoV-2 vaccination, including hypertension (ICD-10: I10), cancer (ICD-10: I10), diabetes mellitus (ICD-10: E10–E14), obesity (ICD-10: E66), chronic heart disease (ICD-10: I20–I25, I48, I50), chronic bronchitis, asthma or chronic obstructive lung disease (COPD) (ICD-10: J42–J46), depression (ICD-10: F32, F33), anxiety disorders (ICD-10: F41), somatoform disorder (ICD-10: F45), and dementia (ICD-10: F01–F03, G30). Due to the large sample size, *p*-values < 0.01 were considered statistically significant. Analyses were carried out using SAS version 9.4 (SAS institute, Cary, NC, USA).

## 3. Results

### 3.1. Basic Characteristics of the Study Sample

The present study included 531,468 individuals who received a total of 908,869 SARS-CoV-2 vaccinations between April and September 2021. The mean age (SD) was 53.9 years (18.2); 49.4% were women. The most-common previously documented comorbidities of patients receiving a SARS-CoV-2 vaccination were hypertension (32.5%), diabetes mellitus (14.1%), depression (14.4%), chronic heart diseases (13.4%), and chronic respiratory tract disease (12.9%). The basic characteristics of the study cohort are displayed in Table 1.

### 3.2. Frequency of Vaccination-Associated Side Effects Reported by Patients

In total, 25,483 patients reported side effects for 28,287 (3.1%) vaccinations. On average, side effects were reported two days after vaccination. The frequency of reported side effects was highest among younger individuals and decreased gradually with age from 5.3% in the group aged between 18 and 30 years to just 1.7% in the age group above 70 years. Side effects were coded more often in men (3.3%) than in women (2.9%). Of all 28,287 documented adverse reactions (ICD-10: U12.9), the specific symptom responsible for coding an adverse reaction was reported in only 12,575 cases. Of these 12,575 cases, pain in limb (ICD-10: M79.6, 24.3%) occurred most frequently, followed by fatigue (ICD-10: R53.0, 21.0%), dizziness (ICD-10: R42.0, 17.9%), joint pain (ICD-10: M25.5, 15.7%), fever (ICD-10: R50.9, 9.5%), nausea (R.11.0, 7.5%), and myalgia (ID-10: M79.1, 6.4%).

### 3.3. Association between Age, Sex, Comorbidities, and Side Effects Reported following Vaccination against SARS-CoV-2

Finally, we performed multivariate regression analyses to further break down the potential factors associated with an increased diagnosis of vaccination-related side effects. Here, young age was associated with much higher odds of reported side effects (OR: 4.45 in the age group between 18–30 years, OR: 3.50 for 31–40 years, OR: 2.89 for 41–50 years as compared with the age group > 70 years, Table 2). In addition, several comorbidities were found to have a strong association with reported side effects following vaccination, with pre-existing dementia (OR: 1.54), somatoform disorder (OR: 1.53), anxiety disorder (OR: 1.43), depression (OR: 1.37), chronic respiratory tract disease (OR: 1.27), hypertension (OR: 1.20), and obesity (1.14) significantly increasing the odds of reported side effects (Table 2). By contrast, pre-existing cancer, chronic heart disease, or diabetes mellitus were not significantly associated with vaccination-related side effects. Finally, male sex was associated with increased odds of reported side effects (OR: 1.17, Table 2).

## 4. Discussion

The analyses available from randomized clinical trials on SARS-CoV-2 vaccine efficacy and tolerability indicate an extremely positive benefit–risk profile [2,3,4,5]. To complement these analyses, we analyzed 531,468 individuals who received a total of 908,869 SARS-CoV-2 vaccinations in general practices in Germany between April and September 2021 in order to qualitatively analyze the extent to which the general population is affected by documented side effects and identify possible factors associated with the reporting of side effects. In total, 3.1% of patients reported side effects, which they felt warranted a renewed visit to the vaccinating physician or which were actively asked about and documented by the family doctor. This rate of side effects is low compared to that indicated by data from randomized trials, in which adverse events were reported in 21 to 40.6% of patients [2,3,4]. However, the different approaches for assessing side effects cannot be compared systematically since the recording of side effects in our study was completely different to that in the existing randomized controlled trials. In addition, the cohorts in our database are completely different to those in previous clinical trials since the latter are conducted for a single brand of vaccine while our study features all vaccines approved for use in Germany. Nonetheless, our analysis represents the clinical reality very well by capturing vaccinations administered in primary care practices, as well as associated adverse events, and may even offer a better reflection of patients’ perceived burden after SARS-CoV-2 vaccination compared to the burden reported in the clinical trial setting.

Non-specific side effects including headache, fatigue, dizziness, and nausea following SARS-CoV-2 vaccination are common, but generally not serious. Recent metanalyses reported a systemic adverse event (AE) in 46.2% and a local AE in 66.7% of all vaccinated patients, with headache, fever, pain, fatigue, and nausea being the most common side effects [12,13]. Similarly, our real-world analysis revealed pain in limb, fatigue, dizziness, joint pain, fever, nausea, and myalgia as the most common clinical problems after SARS-CoV-2 vaccination. The fact that we found significantly lower rates of documented side effects (about 3% of all vaccinations) must be attributed to the different system of recording used; our database only contains side effects that led to a repeat visit—or so we assume—to the general practitioner and were thus perceived by the patients as particularly burdensome and/or relevant. The pathophysiology of non-specific vaccine side effects in particular is subject to controversy, but given the large number of people who have received or will receive a SARS-CoV-2 vaccine, this topic is of particular relevance. Of note, we failed to demonstrate an association between adverse reactions and the presence of neoplastic disease, diabetes mellitus, or heart failure, disease conditions that frequently lead to the recommendation of booster vaccinations. Haas et al. demonstrated that 76.0% of systemic AEs and 24.3% of local AEs after the first vaccination can be attributed to nocebo responses. In this context, our observation that somatoform disorders and anxiety disorders, as well as preexisting depression, are significantly associated with vaccination-related side effects might be of relevance since these data identify an important subgroup of patients who may be particularly sensitive to even mild discomfort after SARS-CoV-2 vaccination and thus require especially strong reassurance on the relative harmlessness of such side effects [14,15,16,17].

In contrast to somatoform disorders, anxiety disorders, and preexisting depression, our analysis found that increasing age was negatively associated with the reporting of side effects after SARS-CoV-2 vaccination, supporting previous findings [12], which might in part be explained by immunological effects [18,19]. Nevertheless, since male sex was also associated with increased odds of reported side effects, we are unable to exclude the possibility that, in addition to immunological effects, the effects observed may also be due to differential reporting or susceptibility to side effects, different experiences of receiving the vaccine, and differences in other social determinants of health or experiences of health. For example, different age groups and sexes may receive different information about vaccinations and their side effects, and may thus potentially have different expectations concerning the occurrence of side effects, which may then also change their perception (“nocebo effect”) as recently discussed in detail [14,15,16,17]. In addition, a sex/gender bias that has previously been reported for several clinical conditions needs to be discussed [20,21,22]. Finally, the existence of less personal benefits from vaccination and more negative experience from lockdown in younger patients could constitute a negative emotional background prone to the nocebo effect after vaccination (which could be experienced as an undeserved punishment among younger patients).

As pointed out, in contrast to most previous data on side effects after SARS-CoV-2 vaccination that are derived predominantly from randomized trials, our study features a large cohort of outpatients that are representative of the sociodemographic situation in Germany and other high-income countries. However, we acknowledge that our study is subject to a number of limitations, which are mainly related to the study design and cannot be avoided. In brief, all diagnoses are coded using ICD-10 codes, which could potentially lead to the misclassification and undercoding of certain diagnoses. However, the statistical validity of the IQVIA Disease Analyzer database used for the analyses in this study has been proven in numerous previous publications [23,24,25]. In addition, there is a lack of data on the socioeconomic status (e.g., education and income) of patients, as well as lifestyle-related risk factors (e.g., smoking, alcohol consumption). Likewise, data on the number of immunocompromised patients within our study cohort is not available, which could yield novel interesting information on the role of the activation of the immune system in the context of vaccination-associated side effects. We cannot distinguish whether the types of side effects and the timing of their documentation are determined by the doctor or the patient. It is possible that many side effects are documented in the patient’s medical records that were not considered sufficient for a diagnosis and were therefore not available for evaluation in this study. Furthermore, the database does not provide information on severe side effects such as e.g., myocarditis. Thus, our study, in contrast to clinical trials, cannot give an unbiased estimation of the total benefit–risk profile of the SARS-CoV-2 vaccination. Moreover, we cannot exclude the possibility that our study is affected by a selection bias in favor of those particularly prone to vaccination-related side effects, who are more likely to present to their GP for vaccination and who may be more likely to develop side effects after vaccination. Finally, we considered individual vaccinations and not vaccinated individuals in our analyses, which might lead to some bias.

In summary, we present data from a large German primary care provider database showing that about 3% of all patients receiving a SARS-CoV-2 vaccination reported systemic or local side effects. Most of these are rather non-specific symptoms, such as headache and fatigue, and have been discussed extensively even on social media and are therefore known to a large number of patients [12]. Patients with preexisting somatoform disorders, anxiety disorders, and depression might be particularly prone to developing these specific widely discussed side effects in the context of nocebo mechanisms such as AE-related anxiety and expectations. Thus, beyond the individual patient, our data might have implications for the organization of educational and information campaigns for SARS-CoV-2 vaccination since they identify a subgroup of patients requiring special consideration. Moreover, they might help to establish a mechanism for the optimal allocation of patients to different vaccination settings, since it seems likely that those patients who are at a particular risk of experiencing SARS-CoV-2 vaccination-related side effects might benefit from intensive counseling, which can certainly be provided better in the family doctor setting than, for example, in large, anonymous vaccination centers.

## Figures and Tables

**Table 1:** Baseline characteristics of the study sample.

Variable	Proportion Affected among Patients with COVID-19 Vaccination (N, %) *n* = 908,689 Vaccinations
Age (mean, SD)	53.9 (18.2)
Age 18–30	123,610 (13.6%)
Age 31–40	101,618 (11.2%)
Age 41–50	121,327 (13.4%)
Age 51–60	198,856 (21.9%)
Age 61–70	197,306 (21.7%)
Age >70	165,972 (18.3%)
Female	448,725 (49.4%)
Male	459,964 (50.6%)
April 2021	119,067 (13.1%)
May 2021	202,346 (22.3%)
June 2021	256,336 (28.2%)
July 2021	185,572 (20.4%)
August 2021	89,196 (9.8%)
September 2021	56,172 (5.1%)
Comorbidities *	
Hypertension	296,616 (32.5%)
Obesity	76,103 (8.4%)
Cancer	61,156 (6.7%)
Chronic heart disease	122,159 (13.4%)
Diabetes mellitus	127,766 (14.1%)
Chronic respiratory tract disease	116,780 (12.9%)
Dementia	15,369 (1.7%)
Depression	130,973 (14.4%)
Anxiety disorder	54,374 (6.0%)
Somatoform disorder	79,653 (8.8%)

Proportions of patients are given in % unless otherwise indicated. SD, standard deviation. * indicates individuals can have more than one of these comorbidities (414,099 (45.6%) have none one of these comorbidities, 195,108 (21.5%) have one comorbidity, 136,786 (15.1%) have two comorbidities, and 162,696 (17.9%) have at least three different comorbidities).

**Table 2:** Association between predefined variables and side effects reported following SARS-CoV-2 vaccination in general practices in Germany (Multivariate logistic regression model).

Variable	Proportion of Individuals with Reported Side Effects (%)	Adjusted Odds Ratio (OR, 99% CI) *	*p*-Value
Age 18–30	5.3	4.45 (4.20–4.70)	<0.001
Age 31–40	4.4	3.50 (3.30–3.7)	<0.001
Age 41–50	3.9	2.89 (2.73–3.07)	<0.001
Age 51–60	3.2	2.20 (2.08–2.29)	<0.001
Age 61–70	1.8	1.13 (1.06–1.21)	<0.001
Age >70	1.7	Reference	
Female	2.9	Reference	
Male	3.3	1.17 (1.14–1.21)	<0.001
Hypertension	2.8	1.20 (1.15–1.25)	<0.001
Obesity	3.7	1.14 (1.08–1.20)	<0.001
Cancer	2.3	0.97 (0.90–1.04)	0.257
Chronic heart disease	2.5	1.06 (1.01–1.12)	0.021
Diabetes mellitus	2.5	0.97 (0.92–1.02)	0.134
Chronic respiratory tract disease	3.7	1.27 (1.22–1.33)	<0.001
Dementia	3.0	1.54 (1.38–1.71)	<0.001
Depression	4.2	1.37 (1.31–1.42)	<0.001
Anxiety disorder	5.0	1.43 (1.36–1.51)	<0.001
Somatoform disorder	4.8	1.53 (1.45–1.60)	<0.001

* Multivariable regression adjusted for age, sex, and comorbidities. The effect of each variable is adjusted for all other variables listed in the table.

## Data Availability

The datasets used and analyzed during the current study are available from the corresponding author on reasonable request.
